# C-752-05. Pharmacologic Interventions to Prevent Amputation in Severe Frostbite: A Systematic Review and Meta-Analysis

**DOI:** 10.1093/jbcr/irag033.018

**Published:** 2026-04-06

**Authors:** Francesco M Egro, Mahi Pachgade, Matteo Angelini, Hilary Y Liu, Jose Antonio Arellano, Asim Ejaz

**Affiliations:** University of Pittsburgh Medical Center, Pittsburgh, PA; Drexel University College of Medicine, Philadelphia, PA; University of Pittsburgh Medical Center, Mercy Burn Center, Pittsburgh, PA; University of Pittsburgh Medical Center, Mercy Burn Center, Pittsburgh, PA; University of Pittsburgh Medical Center, Pittsburgh, PA; University of Pittsburgh, Pittsburgh, PA

## Abstract

**Introduction:**

Frostbite is a serious injury that occurs when body tissues are exposed to freezing temperatures, potentially resulting in amputation. Tissue damage arises from a combination of direct freezing and subsequent inflammation, vasoconstriction, and thrombosis. Thrombolytic agents are an important component of acute-phase treatment. Iloprost has recently received FDA approval for managing acute frostbite. However, pooled and comparative evidence on their effectiveness remains limited. This study aimed to systematically evaluate and compare the impact of thrombolytic agents and iloprost on amputation outcomes in patients with severe frostbite.

**Methods:**

A systematic review and meta-analysis was conducted in accordance with PRISMA guidelines across PubMed, Cochrane Library, EMBASE, and Web of Science. This study was registered on PROSPERO (CRD420250652369). Eligible articles reported on frostbite treatment with either iloprost or thrombolytic agents in 3 or more patients. The primary outcomes were the number of amputated patients and the digital amputation rate.

**Results:**

Of 7875 studies screened, 12 met the inclusion criteria, including 10 dealing with tPA, 2 with iloprost and 1 reporting about both interventions. They examined 779 patients with severe frostbite (grade 2-4), affecting the upper (50.25%) or lower (49.75%) extremities. Patients were predominantly male (81.64%) with a mean age of 41.63 ± 15.38 years. Thrombolytic therapy significantly reduced the risk of digital amputation in severe frostbite (RR = 0.21; 95% CI: 0.14–0.33; *p*<.00001; I^2^ = 0%). Iloprost showed a trend toward reduced digital amputation risk, although heterogeneity was high (RR = 0.09; 95% CI: 0.00–5.04; *p*=.24; I^2^ = 88%).

**Conclusions:**

Amputation is the most feared complication of severe frostbite. Thrombolytic agents emerged as a key acute intervention to prevent tissue loss in eligible patients. Iloprost may contribute to improved amputation outcomes. Additional high-quality comparative studies are needed to determine the most effective strategy for tissue salvage.

**Applicability of Research to Practice:**

Early thrombolytic therapy should be prioritized in eligible severe frostbite cases to reduce amputation risk. Iloprost may be useful when thrombolysis is contraindicated, though its role is less defined. Standardized protocols and timely intervention are key to improving tissue salvage and patient outcomes.

**Funding for the study:**

N/A.

